# Effectiveness of a Comprehensive Health Literacy Consultation Skills Training for Undergraduate Medical Students: A Randomized Controlled Trial

**DOI:** 10.3390/ijerph17010081

**Published:** 2019-12-20

**Authors:** Marise S. Kaper, Sijmen A. Reijneveld, Frank D. van Es, Janine de Zeeuw, Josué Almansa, Jaap A.R. Koot, Andrea F. de Winter

**Affiliations:** 1 Department of Health Sciences, University Medical Center Groningen, University of Groningen, Hanzeplein 1, P.O. Box 30.001, FA10, 9700 RB Groningen, The Netherlands; s.a.reijneveld@umcg.nl (S.A.R.); j.almansa.ortiz@umcg.nl (J.A.); a.f.de.winter@umcg.nl (A.F.d.W.); 2 Faculty of Medical Sciences, University Medical Center Groningen, University of Groningen, Hanzeplein 1, P.O. Box 30.001, FA10, 9700 RB Groningen, The Netherlands; f.d.van.es@umcg.nl (F.D.v.E.); j.de.zeeuw@umcg.nl (J.d.Z.)

**Keywords:** health literacy, medical education, patient-centred communication, shared decision-making, self-management

## Abstract

Comprehensible communication by itself is not sufficient to overcome health literacy related problems. Future doctors need a larger scope of capacities in order to strengthen a patient’s autonomy, participation, and self-management abilities. To date, such comprehensive training-interventions are rarely embedded in curricula, nor systematically evaluated. We assessed whether comprehensive training increased these health literacy competencies, in a randomized controlled trial (RCT), with a waiting list condition. Participants were international undergraduate medical students of a Dutch medical faculty (intervention: 39; control: 40). The 11-h-training-intervention encompassed a health literacy lecture and five interactive small-group sessions to practise gathering information and providing comprehensible information, shared decision-making, and enabling of self-management using role-play and videotaped conversations. We assessed self-reported competencies (knowledge and awareness of health literacy, attitude, self-efficacy, and ability to use patient-centred communication techniques) at baseline, after a five and ten-week follow-up. We compared students’ competencies using multi-level analysis, adjusted for baseline. As validation, we evaluated demonstrated skills in videotaped consultations for a subsample. The group of students who received the training intervention reported significantly greater health literacy competencies, which persisted up to five weeks afterwards. Increase was greatest for providing comprehensible information (B: 1.50; 95% confidence interval, CI 1.15 to 1.84), shared decision-making (B: 1.08; 95% CI 0.60 to 1.55), and self-management (B: 1.21; 95% CI 0.61 to 1.80). Effects regarding demonstrated skills confirmed self-rated competency improvement. This training enhanced a larger scope of health literacy competences and was well received by medical students. Implementation and further evaluation of this training in education and clinical practice can support sustainable health literacy capacity building of future doctors and contribute to better patient empowerment and outcomes of consultations.

## 1. Introduction

For medical doctors, comprehensible communication by itself is not sufficient to overcome inequality and address the prevalent problems of patients with limited health literacy [1]. Medical doctors need a larger scope of capacities to improve understanding, strengthen autonomy, and support self-management of their patients during medical consultations [2,3,4,5]. Limited health literacy implies that people, and in particular, the elderly and those with a lower socio-economic status, have problems with obtaining, comprehending, judging, and applying health information [1]. People with limited health literacy experience worse health outcomes, have higher hospitalization rates, and use less preventive care [6]. Furthermore, as the growing ageing population leads to an increasing prevalence of chronic illnesses, medical doctors must be able to strengthen people’s ability to manage and take responsibility for their own health [7].

By communicating more effectively in medical consultations, medical doctors contribute to improved understanding, adherence to medical treatment, improved health and (further) prevention of health problems [8,9,10]. Future medical doctors, therefore, need to develop adequate health literacy competencies. For this reason, health literacy capacity building should be integrated in medical curricula [11,12]. Four reviews [12,13,14,15] reported that training in health literacy capacity building for undergraduate health care students improves knowledge of health literacy and skills for communicating clearly and comprehensibly with patients. Despite this, those trainings do not address the full scope of health literacy related problems. Such training for undergraduate students often focuses only on comprehension of information related to functional health literacy (the skills to read and write, which are needed for comprehension of information and to function in a health setting) [16]. Training rarely includes enhancing a patient’s autonomy, participation, and self-management abilities in medical consultations [14]. These skills relate to interactive health literacy (the ability to communicate about health information and use this in different circumstances) and critical health literacy (the ability to analyse information and use this in order to control one’s health) [16].

To date, such comprehensive health literacy training-interventions for medical students are rarely embedded in curricula or, to our best knowledge, systematically evaluated in a randomized controlled trial (RCT) with a pre-post design [12]. We, therefore, inserted components of a comprehensive training regarding functional, interactive, and critical health literacy [16,17] into a routinely scheduled medical consultation skills (MCS)-training for students in the pre-clinical training phase.

The routine MCS-training is embedded into the competency-focused medical curriculum based on the CanMEDS framework [18,19]. The focus of competency building is on the development of knowledge, attitudes, and skills. The CanMEDS framework is structured around seven key competencies, which are medical expert, communicator, collaborator, manager, health advocate, scholar, and professional. These competencies were devised to educate and facilitate the functioning of physicians that is effective to meet the needs of patients and influence health care outcomes.

The inserted components were based on a comprehensive health literacy training which was previously developed and evaluated among qualified health professionals [17,20]. Professionals taking part reported that this training increased their competency to enhance understanding of information, involve patients in shared decision-making and enhance the self-management abilities of patients [17,20]. The objective of this study was to assess whether this comprehensive Health Literacy MCS-training increased the health literacy competencies of undergraduate medical students in an RCT, with a waiting list condition.

## 2. Materials and Methods

### 2.1. Design

An RCT with a waiting list condition was performed between April and June 2017. In this RCT, a 5-week training was provided to the group of students in the intervention and to students in the waiting list condition later on. The study included three assessments based on self-report. For a subsample of participants, the self-assessments were validated by video-observation. Study results are reported following CONSORT guidelines [21].

### 2.2. Setting and Participants

The setting was a Dutch medical faculty and in that faculty, the Learning Community Global Health. In this Learning Community, bachelor students follow a curriculum, which consists of two main programs: (1) a competency-focused program based on the CanMEDS framework, and (2) a Causes of Disease program focusing on medical knowledge. We included second-year undergraduate medical students from the Learning Community Global Health. Some students originated from the Netherlands and some from abroad (around 50%), mostly European countries. This sample of students had already participated in three training modules regarding consultation skills training dealing with basic concepts of doctor-patient interaction. Therefore, these students, now in their second year, all met the entry requirements for the Medical Consultation Skills training, which is part of the regular curriculum. The total eligible sample involved 90 second-year medical students who were scheduled for this regular consultation skills training. When students provided informed consent to take part in the research measurements, they were randomly allocated to the intervention group or the waiting-list condition. Eleven students decided not to participate in the study. These students received their regular training in a separate group and did not take part in the research measurements. The study was performed in line with the Declaration of Helsinki, and the independent NVMO-Ethical Review Board (registration number 994) approved the study protocol.

### 2.3. Procedure

Medical students (2nd year) who were scheduled for the Health Literacy MCS-training received written information on the RCT and an invitation to join the study. Students provided written informed consent for self-rated assessments and videotaped consultations. Next, students who participated in the study were randomly allocated to the intervention condition or the waiting-list condition. In the study, students evaluated their self-rated health literacy competency by means of questionnaires. Self-rated health literacy competency was evaluated at three time points: (1) at baseline, before the intervention took place (T1). (2) At five weeks, after the intervention group had received the Health Literacy MCS-training (T2). (3) At ten weeks, after the waiting list condition had received the same Health Literacy MCS-training (T3). The waiting-list condition served as the control group at the measurements T1 and T2 in order to assess the effects of the training by comparing outcomes between both conditions. Next, students in the waiting-list condition, therefore, received the training between measurements T2 and T3, to reach equal competencies at the end of their bachelor. In this way, the educational requirements were met in that the training content and format had to be similar for both groups at the end of this stage.

For a subsample of students, self-reports were validated by video-observation. Two raters assessed the videotaped medical consultations of the participant subsample. The medical consultations were recorded at the second and the sixth training sessions of the students in the intervention condition and in the waiting list condition later on. Reasons for using videotaping of a smaller subsample were the limited availability of time and equipment to record each individual student in session 2. The groups of students with and without videotaped consultations did not significantly differ on self-rated health literacy competency when differences were tested using independent sample *t*-tests.

### 2.4. Intervention

The intervention encompassed the comprehensive Health Literacy MCS-training, which had three core objectives (Table 1): (1) enhancing awareness and health literacy related knowledge. (2) Improving patient-centred communication to facilitate understanding of information, enhancing autonomy, and enabling self-management. (3) Integrating these communication skills. A number of interactive learning strategies were applied to strengthen students consultation skills during simulated medical consultations [12,13,22,23]. The 11-h training lasted five weeks and consisted of six sessions: the first session was a 1-h introductory lecture on health literacy, followed by four weekly 2-h training sessions in small groups of 10 students. The sixth session was a summative oral assessment of a simulated medical consultation, of which the duration was 12 min per student. According to institution guidelines, participants were allowed to miss a maximum of two sessions. Moderators of the training sessions were recently graduated psychologists with a Master’s degree and students following a Master in Psychology. These moderators received 4 h of health literacy training in two sessions. The first session involved instruction on health literacy and the impact of limited health literacy on patients. The second session involved instruction on training health literacy consultation skills and calibration on the observation of such skills.

The components of the health literacy training were integrated in a routine training of medical consultation skills for medical students. A routine training in medical consultation skills is based on a framework with seven stages: (1) becoming acquainted with the patient, (2) exploring the care request, (3) history taking, (4) physical examination, (5) diagnostic, (6) treatment, and (7) closing the consultation [24]. We embedded the Health Literacy MCS-training in the stages 5 to 7 of this framework: comprehensible communication was integrated into stage 5, shared decision-making into stage 6, and enhancing self-management into stage 7.

Students practised health literacy consultation skills in small-group sessions. They did so by means of role-played medical consultations between a physician and a patient with limited health literacy. Each doctor and patient role was played by one student during the training-sessions. Fellow students and the facilitator observed the simulated medical consultations and provided feedback. Students had the opportunity to reflect on their videotaped consultation recorded at the first and the last small-group session (being session 2 and 6). In the sixth session, the consultation skills of students were evaluated in a summative oral assessment. Like in the training sessions, each doctor and patient role was played by one student, in line with routine procedures of the faculty for the bachelor phase. Students were evaluated inside their training group. Ten different standardized scenarios were used for ten different physician-patient couples. The rationale for this approach was that students would learn more by observing different case scenarios. Students had to prepare both the physician scenarios and their patient role before the assessment. Scenarios were assigned following the alphabetical order of the list of names within a training group of ten students. In the master phase, it is educational routine that students practise more frequently with standardized patients who are, for example, actors. Additionally, students routinely communicate with real patients in rotating clinical internships during this phase.

We based the patient-roles on frequently identified health literacy problems [4,6]. Senior educators reviewed the role descriptions. The scenarios involved clinical information on topics relating to reproductive and child health, such as smoking during pregnancy, or parents taking care of a child having febrile convulsion or asthma. Scenarios involved instructions on how to play a limited health literate patient [4,6,25,26]: (1) visit the doctor in a later stadium with increased complaints. (2) Demonstrate limited understanding of medical jargon or complex instructions. (3) Demonstrate limited participation in decision making (e.g., be silent, ask few questions, or be unable to state preferences). (4) Having problems with the judgment of information and with self-management (e.g., not being able to provide the correct medication dose or not feeling able to change behaviour related to smoking).

### 2.5. Primary Study Outcomes

The primary outcome was the perceived health literacy competency of medical students in simulated medical consultations. This was measured as knowledge of health literacy, self-efficacy, and attitude and health literacy consultation skills. We used a self-rated questionnaire to measure health literacy competency (See Supplementary Materials S1 for the questionnaire). The subscales of the questionnaire were selected from research on health literacy education and capacity building [27,28,29,30,31,32]. In case there were no suitable instruments subscales available to measure the required outcomes, the subscales were based on theoretical models on patient-centred communication [27,28,29,30,31,32].

The four subscales of the questionnaire corresponded with the health literacy competencies. The reliability of the four categories in the questionnaires was adequate, with Cronbach’s alphas ranging from 0.701 to 0.915. The four subscales were:

(1) “Knowledge of Health literacy [27,31,32]” had six items [27] rated on 7 points ranging from “strongly disagree” to “strongly agree”.
(2) “Health Literacy Attitude” had four items rated on 7 points ranging from “strongly disagree” to “strongly agree”. This scale was based on the Health Literacy Strategies Behavioural Intention Questionnaire (HLSBI) [33,34], with a calculated Cronbach’s alpha of 0.76 (reliability is adequate) [33,34].
(3) “Self-efficacy” to apply consultation skills had 9 items [35] rated on 5 points ranging from “not at all confident” to “very confident”.
(4) “Health literacy consultation skills [36,37]” had 16 items with questions on four different skills rated on 7 points ranging from “never” to “every time”: “gathering information” (4 items) [17], “providing information” (5 items) [27,31,32], “shared decision-making” [30] (4 items), and enabling self-management” (3 items) [37].

### 2.6. Validation of Primary Outcomes

As students have sometimes been shown to overestimate their own skills [38], we validated the self-rated primary outcomes by comparing them with observed health literacy consultation skills. To investigate these, we observed video-taped consultations, which were coded following the scheme of the Four Habits [39]. The items of the Four Habits matched with the health literacy consultation skills of the training. Scales of the Four Habits involved [39]: (1) ‘Invest in the beginning’ (6 items), (2) ‘Eliciting the perspective of patients’ (3 items), (3) ‘Demonstration of empathy (4 items), (4) ‘Investing in the end of the consultation’ (10 items). Three specific items were added to Habit 4 on ‘enhancing self-management’ (See Supplementary Materials S2 for the video-observation form). The Four Habits coding scheme was reported to have sufficient reliability and correlated significantly with scales in the Roter Interaction Analysis System [39]. Each item is supposed to be rated on a 5-point scale, with anchors one, three, and five described in specific behavioural terms.

The rating of the observed skills in the videotaped consultations was conducted in two steps. First, MK and CvZ independently rated a pilot sample of five videos. They agreed on some adaptations to make the checklist more specific and about when to code certain behaviours as applicable. Second, MK and CvZ independently rated the complete video subsample. Their ratings were mostly consistent or had a maximum difference of 1 point. The items ‘engage in small talk (H1C)’ and ‘impact on life (H2C)’ [39] were mostly rated as not applicable and were excluded from the analysis. The mean intra-class correlation between the ratings of the four habits for session two was 0.510 and for session six 0.609, indicating acceptable interrater reliability. The combined mean ratings of MK and CvZ were used for further analysis.

### 2.7. Demographic Variables

Demographic variables were measured at baseline (T1). Variables concerned gender, age, nationality, prior education, and confidence in participating in role-played medical consultations (rated on 5 points ranging from ‘not at all confident’ to very confident’). Immediately after their training, participants filled in six evaluation questions [28] rated on 7 points ranging from ‘strongly disagree’ to 7 = ‘strongly agree’.

### 2.8. Sample Size

The power analysis showed that 74 participants were needed (n = 37 per group) in order to find a mean difference of 1 point between the intervention and the control condition for each subscale [27,31] (with the standard deviation being 1.5 points for each of the mean scores within both groups). The alpha level was set to 0.05, and the power was set to 0.80. Following the power analysis, the requirement was 98 students in order to account for a dropout rate of 33% in the follow-up measurements.

### 2.9. Randomization and Blinding

Participants were randomized with equal probability over the two conditions using a computer-generated algorithm. The researchers, moderators, and students could not be blinded for the allocation because they were aware of when the training-sessions were scheduled.

### 2.10. Analysis and Reporting

First, we described the participant flow in a diagram. Second, we assessed if the intervention and control group differed regarding demographic variables. Depending on the measurement level of the variable, we assessed differences with either Chi-square tests or independent sample *t*-tests. Third, we assessed if the intervention and control group differed regarding the various outcome measures related to the health literacy competencies. Our outcome variables (i.e., the subscales knowledge, attitude, and self-efficacy and consultation skills) had continuous measurement levels. Each of them consisted of the mean scores of responses on several questions, which were rated on a 7-points rating scale, except forself-efficacy, which was rated at a 5-points scale. For every subscale, the mean scores were calculated by counting the total sum divided by the number of items. Next, before we conducted the multi-level analyses, we assessed the outcome variables, which were normally distributed. We also checked the residuals of the multilevel models, which showed that all followed a normal distribution. The intervention effect was estimated using multi-level analysis (measurements at T2 and T3 are nested within students), comparing changes in the outcome variables between measurements at T2 and T3 across groups, adjusted for baseline (T1). The treatment effects were estimated based on intention to treat. Statistical significance of treatment effects was assessed as an alpha of 0.05. We imputed mean scores for four participants, each of whom had at maximum 50% of missing questions in maximum one subscale. Fourth, as validation of self-rated assessments, changes were measured in observed skills between the first and the last small-group session (being session 2 and 6, as session one was an introductory lecture), which was based on the rated videotaped consultations. The change in observed skills was measured in the subsample of the intervention and control groups after they received the training. We tested statistical significance using paired sample *t*-tests.

## 3. Results

### 3.1. Participant Flow

**Figure 1:**
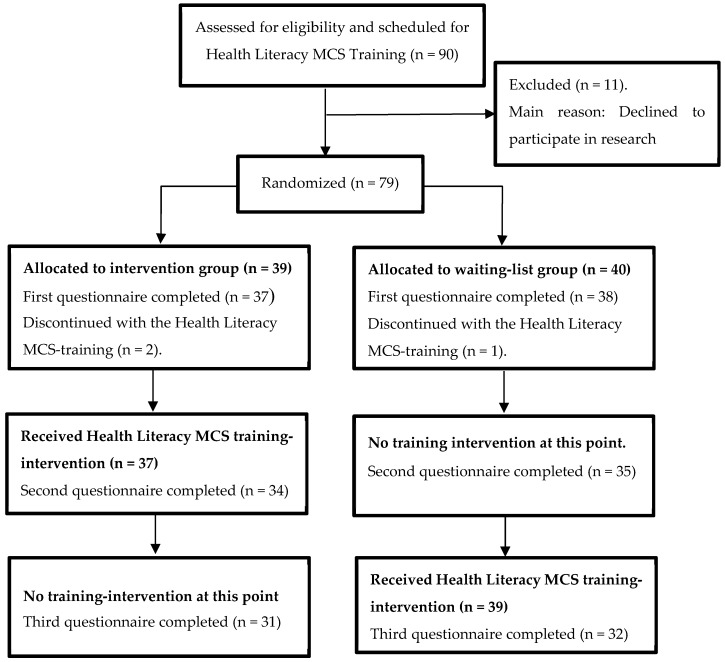
Flow of students throughout the randomized controlled trial (RCT).

The flow of student throughout the RCT is shown in Figure 1. In total, 79 students consented and took part in the study. After randomization, students were allocated either to the intervention group (n = 39) or to the waiting list condition (n = 40). We evaluated self-rated health literacy competency at three time points: (1) at baseline (T1). (2) At five weeks, after the intervention group had received the Health Literacy MCS-training (T2). (3) At ten weeks, after the waiting list condition had received the same training (T3). The waiting-list condition served as the control group at measurements T1 and T2. Next, the training was also provided to students in the waiting-list condition between measurements T2 and T3, to reach that they had equal competencies at the end of their bachelor. In this way, the educational requirements were met that the training content and format had to be similar for both groups at the end of this stage. Eighty percent (n = 63) of the students also gave permission for videotaping of their conversations. For a subsample of in total 24 participants, videotaped consultations (48 in total) of training sessions 2 and 6 were available (intervention group n = 12; waiting list condition n = 12).

In both groups, some students missed training sessions, at a maximum of two. The sessions missed in the intervention group were one session (n = 12) and two sessions (n = 6). Sessions missed in the waiting list group were one session (n = 13) and two sessions (n = 9).

### 3.2. Demographic Variables

Between students in the intervention group and the waiting list group, there were no significant differences related to the demographic variables such as gender, age, prior education, and nationality (Table 2).

### 3.3. Effect of Health Literacy MCS-Training on Primary Outcome

After their training, students reported significantly greater increases in health literacy competency in comparison with the waiting list group. The increases included awareness and health literacy related knowledge, self-efficacy, and consultation skills (Table 3). We found the greatest increases in the skills ‘providing comprehensible information’, ‘shared decision-making’, and ‘supporting self-management’. ‘Attitude’ and ‘gathering information’ did not significantly increase.

Training provided to the waiting list group (between T2 and T3) led to a similar competency increase as in the intervention group (between T1 and T2). Students in the waiting list condition rated their health literacy competency significantly higher at T3 compared to T2, which is indicated by negative parameter estimates for time 2. Also in the waiting list group, change was greatest for ‘health literacy consultation skills’ (B; 95% confidence interval) (−1.08; −1.32 to −0.85), particularly for ‘providing information’ (−1.26; −1.51 to −1.01), ‘shared decision-making’ (−1.16; −1.50 to −0.83) and ‘enhancing self-management’ (−1.37; −1.80 to −0.95). ‘Health literacy knowledge’ (−0.88; −1.12 to −0.64) and self-efficacy’ (−0.81; −1.07 to −0.55) also significantly increased over time. ‘Attitude’ did not change (0.04; −0.47 to 0.55).

### 3.4. Student’s Evaluations of the Training

The majority of students reported being satisfied with the training and agreed that objectives were achieved (N = 63, 96%). Students reported that a good balance existed between theory and practise (N = 41, 62%). They found it useful to practise with a simulation patient (N = 60, 91%) and receive feedback from moderators and peer students (N = 58, 88%).

In addition to the positive evaluations, students provided some recommendations to improve the training. First, they indicated that having conversations with actors as simulation patients would further increase the effectiveness of the training regarding the development of consultation skills and could also provide a more realistic setting. Second, they indicated that practising with students from other groups would also be helpful, for example, with other students who are unfamiliar to the students. Finally, students suggested that physicians could share concrete experiences of their patients with low health literacy or model how they would communicate with such patients.

### 3.5. Validation with Videotaped Conversations

Upon comparison of the skills recorded at the first and the last small-group session (session 2 and 6), the student’s demonstrated significant progress regarding all four habits for health literacy consultation skills, (Table 4).

We noted the greatest progress in Habit 3, ‘demonstrate empathy’ and in specific items related to enabling self-management: ‘formulate personal goals and realistic instructions’ (mean difference = 0.59, SD = 0.47, *p* < 0.001), ‘encourage questions’ (mean difference = 0.65, SD 0.67, *p* < 0.001), and ‘plan for follow-up’ (mean difference = 0.73, SD 0.71, *p* < 0.001).

## 4. Discussion

This RCT assessed whether a comprehensive Health Literacy MCS-training improved the capacities of medical students. Compared to the control condition of no training, we found that this training significantly increased medical students’ health literacy capacities to support patient’s autonomy and self-management abilities in medical consultations, in addition to comprehensible communication. Observed health literacy communication behaviour increased significantly in both conditions, confirming the self-rated increase in competency. The majority of students reported positive evaluations of the training.

This training increased competency to employ a comprehensive approach to health literacy in medical consultations, with the greatest change in ‘shared decision-making’ and ‘self-management’ [17,40,41]. We found a similar competency increase after training the control group. Three factors may have contributed to the effectiveness of this training. First, we trained students in a comprehensive set of skills to address health literacy in medical consultations [14,17]. Second, we used multiple training-sessions with interactive learning strategies to increase students’ interest, knowledge, self-efficacy, and skills [12,35,42,43]. Students practised skills in simulated consultations. They received and applied feedback in preparation for the oral assessment. By acting as both physicians and limited health literate patients, students may have been better able to reflect on both perspectives and their feedback received, which may have increased learning effects. Third, we used moderators competent in providing communication training and gave them an additional health literacy training of four hours. This allowed the moderators to provide didactic instruction, observe skills, and give feedback. The levels regarding the outcomes ‘attitude’ and ‘gathering information’ were already high at the beginning. These outcomes did not increase any further during the training-intervention. An explanation for these high initial levels may be that students had participated in a basic consultation skills training during their first year. This may have contributed to the higher baseline scores of attitude and gathering information [29,40]. In sum, the Health Literacy MCS-training increased students’ competencies. Both the interactive training format and feedback by competent moderators may have strengthened the health literacy competencies of students.

The significant progress in observed skills on all four habits is in line with observed progress in comprehensible health literacy communication among medical students [44], residents [28] and pharmacy students [41]. Moreover, the congruency that we found in observed skills confirms the validity of the self-rated assessments of health literacy competency. By means of an RCT, we demonstrated that this comprehensive Health Literacy MCS-training was effective among an international sample of medical students. Other studies also successfully implemented health literacy training in curricula for undergraduate medical students using an experiential learning format, although these training programs were less comprehensive [32,45]. We, therefore, expect that it is feasible to embed this comprehensive health literacy training in other medical curricula that provide basic training in consultation skills. In that case, the positive outcomes regarding health literacy competencies are likely to be confirmed.

This comprehensive training among medical students enhanced a broader scope of health literacy competencies, in addition to comprehensible communication. These improved competencies may help future doctors to communicate more effectively during medical consultations and to support the empowerment of their patients. Our findings add to previous studies showing training of medical undergraduate students to be effective regarding certain separate competencies that are relevant for future medical practise, such as increasing knowledge of health literacy and skills in comprehensible communication [12,13,14,15]. However, neither of these studies comprehensively addressed all competencies required to adequately handle the problems in providing quality care to low-health literate patients. A reason for that may be that a full inventory of these competencies was lacking until recently [17,20]. Our findings next show that one training can indeed address this range of competencies, in particular, those of the CanMEDS role of Health Advocate on top of those of the role Communicator. Implementation of this training can thus support sustainable health literacy capacity building of future doctors and contribute to higher patient empowerment and better outcomes of consultations.

### 4.1. Strengths and Limitations

A first strength of this study is its randomized design with a waiting list condition. This randomized design reduced potential bias in the assessment of effects by estimation of the differences between the intervention and control condition. Additionally, we also found that the self-rated health literacy competencies increased in the waiting list group when we also offered the training-intervention to this group later on. This increase confirmed the effects found in the intervention condition during the assessment of effectiveness. A second strength is the analysis of observed skills in videotaped conversations. This enabled validation of the self-reported assessments.

Some limitations should, however, be noted. First, because we could not blind researchers, moderators and students there could be information bias. Effects in the self-rated outcomes could have been overestimated because students perceived their skills improved over time, or from a learning effect regarding the rating of their perceived competencies. Compared to the observation of skills, studies have shown that students tend to overestimate their level of self-rated consultation skills indeed [38]. This is why we added observations of students’ skills demonstrated in videotaped conversations in order to formally validate the assessment of training outcomes. The findings on these observations showed that the effect of such an overestimation was limited according to these formal assessments.

Second, we could only observe the behaviour of a subsample of twenty-four students, i.e., one-third of the entire sample. However, participants with and without videotaped consultations did not differ in self-rated health literacy competency. Third, the use of medical students as simulated patients during the training may have led to bias regarding the development of consultation skills. However, the use of students as simulated patients is routine practise in most bachelor phases of medical faculties because of costs and because students learn by simulating being a patient themselves. Fourth, to some extent, self-reported skills may have been overestimated, when compared to the general observation [38]. Fifth, we did not conduct follow-up research in the clinical phase of medical education and investigate the effects of health literacy training among medical residents and their patients [12].

### 4.2. Implications

This RCT showed a comprehensive training to be very effective in increasing health literacy competency and had features allowing an easy embedding in many undergraduate medical curricula. This evidently calls for its wider implementation in various curricula for future health professionals. Integration of this training in other medical curricula is feasible, particularly if a curriculum already offers basic training in competency-focused consultation skills. The effectiveness of the training could be enhanced when medical students have the opportunity to interact with actors as standardized patients, which also could provide a more realistic setting. Second physicians could share concrete experiences of their patients with low health literacy or model how they would communicate with such patients. Extension of the training during the clinical phase could, for example, address health literacy capacities among more specific patient categories with complex problems, and fluent use of patient-centred communication skills to influence behaviour change.

Future research should evaluate outcomes of comprehensive health literacy training among student samples from other medical and health care institutions and in country-specific contexts. Long-term follow-up studies are also required to determine the sustainability of training outcomes in the clinical phase [12,32]. Research should investigate the effects of the educational intervention on the perceived and observed skills of medical residents and examine outcomes among patients being, for example, content with consultations, the informed health decisions, and adherence related to medical treatment.

## 5. Conclusions

In this RCT, we found that this effective training increased a broader scope of health literacy competencies of undergraduate medical students. In particular, these regarded skills to support empowerment and self-management abilities of patients, in addition to comprehensible communication. The training was well received by medical students. Comprehensive health literacy training, unlike training focusing only on functional health literacy, particularly strengthens the skills of medical students to enhance the more complex communicative and critical health literacy levels. Wider implementation of the effective educational intervention in clinical education and practice can support sustainable health literacy capacity building of future doctors and contribute to higher patient empowerment and better outcomes of consultations.

## Figures and Tables

**Table 1:** Outline of the health literacy-medical consultation skills (MCS) training-intervention.

Overview of Sessions	Learning Outcomes on Health Literacy Competencies
1. Introduction lecture (1 h). Outline of Health Literacy MCS training	Distinguish health literacy levels among people. - Define functional, communicative, and critical health literacy skills. - Describe the impact and frequency of problems related to health literacy. - Define methods to examine health literacy levels.
2. Diagnostic consultation (2 h) Announce diagnosis and provide understandable information connected to care request. Informing and anticipating of emotions and questions.	Demonstrate skills to enhance functional health literacy: - Asking open and easy questions to facilitate gathering of information. - Be able to examine the level of health literacy. - Prioritize and provide information to patients that matches with their acquired health literacy skills. - Use plain language, with avoidance of jargon.
	- Acknowledge emotions, concerns, and feelings of shame. - Encourage patients to ask questions. - Use teach-back to check patients’ comprehension and insight into the diagnosis and reteach information if needed.
3. Treatment consultation (2 h). Advise on treatment options and provide understandable information. Deliberate pros and cons of treatment options.	Demonstrate skills to address interactive health literacy: - Stimulate patients to take part in shared decision-making. - Teach patients to express their concerns and ask their questions. - Clarify treatment possibilities: check prior knowledge, give brief, understandable information on treatment, and discuss harms and benefits.
	- Facilitate patient to consider pros and cons in decisions. - Offer support to explore individual considerations.
4. Closing consultation (2 h). Clear instructions on treatment Closing, arrange of follow-up and end consultation.	Demonstrate skills to address critical health literacy: - Discuss strategies to prepare for self-management. - Incorporate patients’ perspectives to enable self-management; - Investigate if patients are willing to adapt their behaviour and obstacles to treatment adherence: formulate personal goals. - Give easy-to-understand instructions that match with previous knowledge. - Strengthen self-efficacy of people.
	- Use teach-back to check patients’ comprehension and insight into the medical treatment and reteach if needed. - Discuss the process for follow-up related to checking of self-care and repeating instructions on medical treatment. - Investigate need and possibilities for aid provided by from social networks or professionals.
5. Integration of consultation skills (2 h)	Demonstrate and evaluate own use of communication skills to address health literacy.
6. Summative oral assessment of consultation skills (2 h).	Demonstrate and evaluate own use of communication skills to address health literacy.

**Table 2:** Demographic variables (T1) of students by group before the intervention.

Demographic Variables	Intervention Group	Control Group	*p* Value
Age, mean (SD) ^1^	21.22 (1.96)	21.41 (2.31)	0.69
Gender (female), *N* (%) ^2^	27 (75.0%)	26 (66.7%)	0.43
Prior education, *N* (%)			0.36
- Dutch High school	14 (37.8%)	13 (33.3%)	
- Foreign education	19 (51.4%)	17 (43.6%)	
- Other	4 (10.8%)	9 (23.1%)	
Nationality, *N* (%)			0.84
- Netherlands	17 (45.9%)	17 (43.6%)	
- Other countries	20 (54.1%)	22 (56.4%)	
Confidence in use of skills in role-play with patients, mean (SD) ^3^	3.49 (1.12)	3.79 (0.77)	0.17

^1^*p* value from independent *t*-test. ^2^ *p* value from Chi-square test. ^3^ Rated on a five-point scale: 1 = ‘not at all confident’ to 5 = ‘very confident’.

**Table 3:** Mean scores of primary outcomes at T1, T2, and T3, and differences between intervention and control condition regarding change from T1 to T2 (‘intervention effect’).

Primary Outcome Variables	Group ^1^	T1	T2	T3	Intervention Effect	
		Mean (SD)	Mean (SD)	Mean (SD)	B (95% CI) ^2^	*p*
HL knowledge	I	4.54 (0.69)	5.38 (0.64)	5.47 (0.57)	0.81 (0.47; 1.15)	<0.001
	C	4.90 (0.80)	4.88 (0.73)	5.79 (0.54)		
Self-efficacy in HL consultation skills	I	3.13 (0.71)	3.68 (0.63)	3.84 (0.68)	0.68 (0.32; 1.04)	<0.001
	C	3.31 (0.78)	3.36 (0.64)	4.17 (0.36)		
Attitude towards HL consultation	I	5.96 (0.98)	5.82 (1.16)	5.72 (1.24)	0.06 (−0.65; 0.78)	0.860
	C	5.75 (1.29)	6.03 (0.89)	6.20 (1.04)		
Total HL consultation skills	I	4.61 (0.73)	5.42 (0.73)	5.50 (0.63)	1.04 (0.70; 1.37)	<0.001
	C	5.03 (0.70)	4.71 (0.77)	5.82 (0.69)		
Of which per consultation skill:						
- Gathering information	I	5.47 (0.57)	5.59 (0.92)	5.91 (0.71)	0.29 (−0.08; 0.65)	0.120
	C	6.05 (0.75)	5.50 (0.88)	6.09 (0.65)		
- Providing information	I	4.26 (0.79)	5.64 (0.70)	5.43 (0.56)	1.50 (1.15; 1.84)	<0.001
	C	4.65 (0.74)	4.37 (0.71)	5.68 (0.74)		
- Shared decision making	I	4.59 (0.95)	5.28 (0.92)	5.41 (0.92)	1.08 (0.60; 1.55)	<0.001
	C	5.11 (1.05)	4.79 (0.94)	5.97 (0.75)		
- Enabling self-management	I	4.08 (1.23)	5.01 (0.99)	5.20 (0.94)	1.21 (0.61; 1.80)	<0.001
	C	4.22 (1.40)	4.13 (1.33)	5.49 (1.15)		

^1^ I = intervention group; C = is the control group or waiting list condition. ^2^ B = parameter estimate of differences T2 compared to T3 between intervention and control groups, adjusted by T1; CI = confidence interval of the multi-level analyses. Competency ratings corresponded with the particular subscales. In each subscale, the mean scores were calculated by counting the total sum divided by the number of questions. (See Methods Section 2.5).

**Table 4:** Comparison of observed mean scores of videotaped consultations of the first and the last small-group training session (session 2 and 6).

Habits ^4^	Session 2	Session 6	Mean Difference	*p ^3^*
	Mean (SD) ^1^	Mean (SD)	Mean Diff. (95% CI) ^2^	
1. Invest in the beginning (6 items).	3.53 (0.25)	3.83 (0.38)	0.30 (0.15; 0.45)	<0.001
2. Eliciting perspective of patients (3 items).	3.52 (0.38)	3.74 (0.43)	0.22 (0.14; 0.42)	0.040
3. Demonstration of empathy (4 items)	3.27 (0.33)	3.69 (0.46)	0.42 (0.25; 0.59)	<0.001
4. Investment in the end of consultations. (10 items)	3.35 (0.25)	3.73 (0.31)	0.38 (0.26; 0.50)	<0.001

^1^ M = Mean; SD = standard deviation. ^2^ CI = confidence interval. *^3^ p =* paired-samples *t*-test. ^4^ Rating of items took place on a 5 points scale, with points one, three and five described in specific behavioural terms.

## Supplementary Materials

The following are available online at https://www.mdpi.com/1660-4601/17/1/81/s1. S1. Questionnaires on Health literacy consultation skills: before and after education. S2. Video-observation form.
