# Supplementary material for: Effectiveness of a Comprehensive Health Literacy Consultation Skills Training for Undergraduate Medical Students: A Randomized Controlled Trial
Source: Int J Environ Res Public Health. 2019 Dec 20;17(1):81. doi: 10.3390/ijerph17010081 (PMC6982343; doi:10.3390/ijerph17010081)
Supplement: Supplementary file 1 [file ijerph-17-00081-s001.zip › S1 Questionnaire HL consultation.docx]

**S1. Questionnaires on Health literacy consultation skills:**

**before and after education**

**Questionnaire 1.**

**Health Literacy Consultation skills - before education**

**Questionnaire 2**

**Health Literacy Consultation skills - after education**

# Questionnaire 1:

# Your health literacy consultation skills before training

| Your unique participant number: |  |
| --- | --- |

**INTRODUCTION**

Dear Student,

We ask you to fill in this first questionnaire because you gave permission to participate in the study: “*How can you address low health literacy in Medical Consultations?”.* All medical students in the 2^nd^ year from the LC Global Health participate in the Medical Consultation Skills training in semester 2.2. In this training, we integrated consultation skills to address low health literacy problems. You will receive study credits based on your participation in the Medical Consultation Skills Training. These credits are not dependent on your participation in the study.

European research has found that nearly 50% of people have low health literacy. People with low health literacy do not have the skills necessary to find, understand or use information about health. As a medical student, you have the potential to address problems of patients with low health literacy during medical consultations in order to promote their health outcomes. For this reason we designed the Medical Consultation Skills Training to strengthen your knowledge and skills in addressing low health literacy of patients during simulated medical consultations.

In this study linked to Medical Consultation Skills Training we want to know if knowledge and skills of students’ improve after following this training. We developed this questionnaire to study the quality of communication between medical students and patients with low health literacy in simulated roleplays.

We ask you to fill out this questionnaire three times: 1) before the start of the Medical Consultation Skills Training Training, 2) immediately following the training, and 3) two months after the training. You received this questionnaire with your unique respondent number, in order to compare your answers provided in the three questionnaires. Your answers are confidential and will be saved under your unique number. Answers will not be linked to your name or student number. For the quality of the research we use different questionnaires. Because of this, some questions may look similar and some response scales differ from each other. It takes about 15 minutes to answer the questions. Please note questions are printed on both sides! The topics of the questions are:

1. General questions
2. Your knowledge of health literacy
3. Your consultation skills focused on health literacy
4. Your opinion on using health literacy consultation skills (attitude)
5. Your confidence in using health literacy consultation skills

If you have any questions related to the study and/or the Medical Consultations Skills Training please contact our researcher Marise Kaper, email: [m.s.kaper@umcg.nl](mailto:m.s.kaper@umcg.nl) – telephone: 050-3616968.

With kind regards,

Jaap Koot & Marise Kaper

Dean LC Global Health Researcher Health Sciences

1. **General questions**

| 1. What is your age in years? | ……………. years |
| --- | --- |
| 1. What is your sex? | - Male - Female |
| 1. Have you previously received training on health literacy? | - 1 – Never - 2 – Rarely - 3 – Occasionally - 4 – Regularly - 5 – Very often |
| 1. How confident are you to use communication skills in roleplays with patients? | - 1 – Not at all confident - 2 – Slightly confident - 3 – Neutral - 4 – Moderately confident - 5 – Very confident |

**B. Your knowledge about health literacy**

*Please indicate how much you agree or disagree with the following questions. Choose only one answer.*

| **Knowledge** | **1**  **Strongly disagree** | **2**  **Dis-agree** | **3**  **Some-**  **what disagree** | **4**  **Neither agree nor disagree** | **5**  **Some-what agree** | **6**  **Agree** | **7**  **Strongly agree** |
| --- | --- | --- | --- | --- | --- | --- | --- |
| 1. I understand what it means for patients to have low health literacy |  |  |  |  |  |  |  |
| 1. I know the prevalence of low health literacy |  |  |  |  |  |  |  |
| 1. I know the groups that are more likely to be low health literate |  |  |  |  |  |  |  |
| 1. I understand the health outcomes associated with low health literacy |  |  |  |  |  |  |  |
| 1. I do a good job identifying low health literate people. |  |  |  |  |  |  |  |
| 1. I am good at knowing whether or not patients understand what I tell them. |  |  |  |  |  |  |  |

**C. Your consultation skills focused on health literacy**

*The following medical consultation and teaching skills have been described as effective with patients with low health literacy. In your first and second year you practiced medical consultation skills in roleplays with simulated patients. We want to know to which extent you practiced these health literacy consultation skills in prior training. It is also possible that you never practiced the skills.*

*Therefore, please indicate how frequently you used the following health literacy consultation skills during roleplay conversations with simulated patients. Choose only one answer.*

| **Gathering information** | **1**  **Never** | **2**  **Rarely** | **3**  **Occasio-nally** | **4**  **Some-times** | **5**  **Fre-quently** | **6**  **Usually** | **7**  **Every time** |
| --- | --- | --- | --- | --- | --- | --- | --- |
| 1. asking open questions |  |  |  |  |  |  |  |
| 1. use active listening techniques to gather information |  |  |  |  |  |  |  |
| 1. Observe cues related to non-verbal communication |  |  |  |  |  |  |  |
| 1. Create a shame-free environment |  |  |  |  |  |  |  |
| **Providing information** | **1**  **Never** | **2**  **Rarely** | **3**  **Occasio-nally** | **4**  **Some-times** | **5**  **Fre-quently** | **6**  **Usually** | **7**  **Every time** |
| 1. Speaking slowly |  |  |  |  |  |  |  |
| 1. Using plain, non-medical language |  |  |  |  |  |  |  |
| 1. Show or draw pictures |  |  |  |  |  |  |  |
| 1. Limit the amount of information provided and repeat it |  |  |  |  |  |  |  |
| 1. Use teach-back   *(let the patient tell information in their own words to check understanding).* |  |  |  |  |  |  |  |
| **Shared decision making** | **1**  **Never** | **2**  **Rarely** | **3**  **Occasio-nally** | **4**  **Some-times** | **5**  **Fre-quently** | **6**  **Usually** | **7**  **Every time** |
| 1. Make patients aware that they have a choice in health care or treatment. |  |  |  |  |  |  |  |
| 1. Inform patients about health care or treatment options in more detail. |  |  |  |  |  |  |  |
| 1. Support patients to explore ‘what matters most to them’ after informing on treatment options. |  |  |  |  |  |  |  |
| 1. Train patients to participate in shared decision making. |  |  |  |  |  |  |  |
| **Enabling self-management** | **1**  **Never** | **2**  **Rarely** | **3**  **Occasio-nally** | **4**  **Some-times** | **5**  **Fre-quently** | **6**  **Usually** | **7**  **Every time** |
| 1. Assess barriers and facilitators relating to treatment compliance. |  |  |  |  |  |  |  |
| 1. Involve the patient in formulating personalized goals and action plans. |  |  |  |  |  |  |  |
| 1. Train patients to perform adequate behaviour to manage their own health. |  |  |  |  |  |  |  |
| *In the previous questions 15 to 30 you indicated how frequently you used each medical consultation skill in roleplay with simulated patients.*   1. Which of these consultation skills do you find **easy** **to apply** in roleplay with a simulation patient? Explain your answer: | | | | | | | |
| *In the previous questions 15 to 30 you indicated how frequently you used each medical consultation skill in roleplay with simulated patients.*   1. Which of these consultation skills, do you find **difficult to apply** in roleplay with a simulation patient? Explain your answer: | | | | | | | |

**D. Your opinion on using health literacy consultation skills (attitude)**

*Health literacy consultation skills are defined as the communication and teaching strategies that have been described as effective with low health literacy patients. These include, plain language communication, which is the avoidance of medical jargon, and Teach-Back, which is a teaching strategy that has the patient teach back to the provider the information just presented to them and also include skills related to shared decision making and promoting self-management. Please read each question and circle the answer that best reflects your opinion on the use of health literacy consultation skills. Choose only one answer.*

| **Questions** | **Scale** | | | | | | |
| --- | --- | --- | --- | --- | --- | --- | --- |
| 1. My use of health literacy consultation skills with patients will result in patients having a better understanding of their illness and its treatment. | 1  Likely | 2 | 3 | 4 | 5 | 6 | 7  Unlikely |
| 1. Improved patient understanding will improve patient outcomes. | 1  Agree | 2 | 3 | 4 | 5 | 6 | 7  Disagree |
| 1. Use of health literacy consultation skills with patients would help patients stay healthy. | 1  Agree | 2 | 3 | 4 | 5 | 6 | 7  Disagree |
| 1. My use of health literacy consultation skills with patients would be a… | 1  Bad idea | 2 | 3 | 4 | 5 | 6 | 7  Good idea |

**E. Your confidence in using health literacy consultation skills**

*With respect to the following questions, please indicate your level of confidence in using health literacy consultation skills in roleplay conversations with simulation patients. Choose only one answer.*

| **How confident are you in your ability to:** | **1**  **Not at all confident** | **2**  **Slightly confident** | **3**  **Neutral** | **4**  **Moderately confident** | **5**  **Very confident** |
| --- | --- | --- | --- | --- | --- |
| 1. Use instruments to identify patients with low health literacy |  |  |  |  |  |
| 1. Identify behaviors typically exhibited by people with low health literacy |  |  |  |  |  |
| 1. Gather information from patients with low health literacy |  |  |  |  |  |
| 1. Provide clear information to patients with low health literacy |  |  |  |  |  |
| 1. Use the teach back or show me technique to check understanding of patients with low health literacy |  |  |  |  |  |
| 1. Create a shame free environment for patients with low health literacy |  |  |  |  |  |
| 1. Involve patients with low health literacy in shared decision making |  |  |  |  |  |
| 1. Train patients with low health literacy to participate in shared decision making |  |  |  |  |  |
| 1. Stimulate patients with low health literacy to manage their own health |  |  |  |  |  |

| 1. Do you have questions and/or comments? |
| --- |

**Thank you very much for your cooperation!**

# Questionnaire 2

# Your health literacy consultation skills after education

| Fill in your unique participant number: |  |
| --- | --- |

**INTRODUCTION**

European research has found that nearly 50% of people have low health literacy. People with low health literacy do not have the skills necessary to find, understand or use information about health.

This training is designed to enhance your competency in addressing low health literacy of patients during simulated medical consultations. It is important for us to know how effective it is.

This questionnaire was therefore developed to study the quality of communication between medical students and patients with low health literacy in simulated roleplays. For the quality of the research we use different questionnaires. Because of this, some questions may look alike.

Please fill out this questionnaire two times: immediately following the training, and two months after the training. It takes about 15 minutes to answer the questions.

The topics of the questions are:

1. Your knowledge of health literacy
2. Your consultation skills focused on health literacy
3. Your opinion on using health literacy consultation skills (attitude)
4. Your confidence in using health literacy consultation skills
5. Your evaluation of the health literacy consultation skills training
6. **Your knowledge of health literacy**

*Please indicate how much you agree or disagree with the following questions. Choose only one answer.*

| **Knowledge** | **1**  **Strongly disagree** | **2**  **Dis-agree** | **3**  **Some-**  **what disagree** | **4**  **Neither agree nor disagree** | **5**  **Some-what agree** | **6**  **Agree** | **7**  **Strongly agree** |
| --- | --- | --- | --- | --- | --- | --- | --- |
| 1. I understand what it means for patients to have low health literacy |  |  |  |  |  |  |  |
| 1. I know the prevalence of low health literacy |  |  |  |  |  |  |  |
| 1. I know the groups that are more likely to be low health literate |  |  |  |  |  |  |  |
| 1. I understand the health outcomes associated with low health literacy |  |  |  |  |  |  |  |
| 1. I do a good job identifying low health literate people. |  |  |  |  |  |  |  |
| 1. I am good at knowing whether or not patients understand what I tell them. |  |  |  |  |  |  |  |

1. **Your consultation skills focused on health literacy**

*The following medical consultation and teaching skills have been described as effective with patients with low health literacy. We want to know to which extent you practiced these health literacy consultation skills. Therefore, please indicate how frequently you used the following health literacy consultation skills during roleplay conversations with simulated patients. Choose only one answer.*

| **Gathering information** | **1**  **Never** | **2**  **Rarely** | **3**  **Occasio-nally** | **4**  **Some-times** | **5**  **Fre-quently** | **6**  **Usually** | **7**  **Every time** |
| --- | --- | --- | --- | --- | --- | --- | --- |
| 1. asking open questions |  |  |  |  |  |  |  |
| 1. use active listening techniques to gather information |  |  |  |  |  |  |  |
| 1. Observe cues related to non-verbal communication |  |  |  |  |  |  |  |
| 1. Create a shame-free environment |  |  |  |  |  |  |  |
| **Providing information** | **1**  **Never** | **2**  **Rarely** | **3**  **Occasio-nally** | **4**  **Some-times** | **5**  **Fre-quently** | **6**  **Usually** | **7**  **Every time** |
| 1. Speaking slowly |  |  |  |  |  |  |  |
| 1. Using plain, non-medical language |  |  |  |  |  |  |  |
| 1. Show or draw pictures |  |  |  |  |  |  |  |
| 1. Limit the amount of information provided and repeat it |  |  |  |  |  |  |  |
| 1. Use teach-back techniques   *(let the patient tell information in their own words to check understanding).* |  |  |  |  |  |  |  |
| **Shared decision making** | **1**  **Never** | **2**  **Rarely** | **3**  **Occasio-nally** | **4**  **Some-times** | **5**  **Fre-quently** | **6**  **Usually** | **7**  **Every time** |
| 1. Make patients aware that they have a choice in health care or treatment. |  |  |  |  |  |  |  |
| 1. Inform patients about health care or treatment options in more detail. |  |  |  |  |  |  |  |
| 1. Support patients to explore ‘what matters most to them’ after informing on treatment options. |  |  |  |  |  |  |  |
| 1. Train patients to participate in shared decision making. |  |  |  |  |  |  |  |
| **Enabling self-management** | **1**  **Never** | **2**  **Rarely** | **3**  **Occasio-nally** | **4**  **Some-times** | **5**  **Fre-quently** | **6**  **Usually** | **7**  **Every time** |
| 1. Assess barriers and facilitators relating to treatment compliance. |  |  |  |  |  |  |  |
| 1. Involve the patient in formulating personalized goals and action plans. |  |  |  |  |  |  |  |
| 1. Train patients to perform adequate behaviour to manage their own health. |  |  |  |  |  |  |  |
| *In the previous questions 11 to 26 you indicated how frequently you used each medical consultation skill in roleplay with simulated patients.*   1. Which of these consultation skills do you find **easy** **to apply** in roleplay with a simulation patient? Explain your answer: 2. Which of these consultation skills, do you find **difficult to apply** in roleplay with a simulation patient? Explain your answer: | | | | | | | |

1. **Your opinion on using health literacy consultation skills (attitude)**

*Health literacy consultation skills are defined as the communication and teaching strategies that have been described as effective with low health literacy patients. These include, plain language communication, which is the avoidance of medical jargon, and Teach-Back, which is a teaching strategy that has the patient teach back to the provider the information just presented to them and also include skills related to shared decision making and promoting self-management. Please read each question and circle the answer that best reflects your opinion on the use of health literacy consultation skills. Choose only one answer.*

| **Questions** | **Scale** | | | | | | |
| --- | --- | --- | --- | --- | --- | --- | --- |
| 1. My use of health literacy consultation skills with patients will result in patients having a better understanding of their illness and its treatment. | 1  Likely | 2 | 3 | 4 | 5 | 6 | 7  Unlikely |
| 1. Improved patient understanding will improve patient outcomes. | 1  Agree | 2 | 3 | 4 | 5 | 6 | 7  Disagree |
| 1. Use of health literacy consultation skills with patients would help patients stay healthy. | 1  Agree | 2 | 3 | 4 | 5 | 6 | 7  Disagree |
| 1. My use of health literacy consultation skills with patients would be a… | 1  Bad idea | 2 | 3 | 4 | 5 | 6 | 7  Good idea |

1. **Your confidence in using health literacy consultation skills**

*With respect to the following questions, please indicate your level of confidence in using health literacy consultation skills in roleplay conversations with simulation patients. Choose only one answer.*

| **How confident are you in your ability to:** | **1**  **Not at all confident** | **2**  **Slightly confident** | **3**  **Neutral** | **4**  **Moderately confident** | **5**  **Very confident** |
| --- | --- | --- | --- | --- | --- |
| 1. Use instruments to identify patients with low health literacy |  |  |  |  |  |
| 1. Identify behaviors typically exhibited by people with low health literacy |  |  |  |  |  |
| 1. Gather information from patients with low health literacy |  |  |  |  |  |
| 1. Provide clear information to patients with low health literacy |  |  |  |  |  |
| 1. Use the teach back or show me technique to check understanding of patients with low health literacy |  |  |  |  |  |
| 1. Create a shame free environment for patients with low health literacy |  |  |  |  |  |
| 1. Involve patients with low health literacy in shared decision making |  |  |  |  |  |
| 1. Train patients with low health literacy to participate in shared decision making |  |  |  |  |  |
| 1. Stimulate patients with low health literacy to manage their own health |  |  |  |  |  |

1. **Your evaluation of the health literacy consultation skills training**

*For each question indicate which answer applies best to you by ticking the appropriate boxes.*

| 1. Have the objectives of this training been achieved according to you? | Yes | Partially | No |
| --- | --- | --- | --- |
| 1. What was the balance between theory and practice (exercises, roleplays, assignments)? | Too much theory | Good | Too much practice |
| 1. Were you sufficiently challenged by the trainer to participate actively? | Yes | Sometimes | No |

*Please indicate how much you agree or disagree with the questions about the health literacy consultation skills training. Choose only one answer.*

| **Questions** | **1**  **Strongly disagree** | **2**  **Dis-agree** | **3**  **Some-**  **what disagree** | **4**  **Neither agree nor disagree** | **5**  **Some-what agree** | **6**  **Agree** | **7**  **Strongly agree** |
| --- | --- | --- | --- | --- | --- | --- | --- |
| 1. The training was tailored to my educational level. |  |  |  |  |  |  |  |
| 1. I found practicing with a simulation patient useful. |  |  |  |  |  |  |  |
| 1. I found the feedback during my roleplay conversations useful. |  |  |  |  |  |  |  |
| 1. I found the visually recording of my roleplay conversations useful. |  |  |  |  |  |  |  |
| 1. I would recommend this education program to other medical students |  |  |  |  |  |  |  |

| 1. What did you learn in this training related to health literacy focused communication? |
| --- |
| 1. Do you have any advice or suggestions related to this training? |

**Thank you very much for your cooperation!**
